# AR12286 Alleviates TGF-β-Related Myofibroblast Transdifferentiation and Reduces Fibrosis after Glaucoma Filtration Surgery

**DOI:** 10.3390/molecules25194422

**Published:** 2020-09-26

**Authors:** Wen-Sheng Cheng, Ching-Long Chen, Jiann-Torng Chen, Le-Tien Lin, Shu-I Pao, Yi-Hao Chen, Da-Wen Lu

**Affiliations:** 1 Graduate Institute of Medical Sciences, National Defense Medical Center, Taipei 114, Taiwan; wnsny@yahoo.com.tw; 2 School of Pharmacy, National Defense Medical Center, Taipei 114, Taiwan; 3 Department of Research and Development, National Defense Medical Center, Taipei 114, Taiwan; 4 Department of Ophthalmology, Tri-Service General Hospital, National Defense Medical Center, Taipei 114, Taiwan; babylong2105@gmail.com (C.-L.C.); jt66chen@gmail.com (J.-T.C.); coolmay01@gmail.com (S.-I.P.); 5 Department of Ophthalmology, Tri-Service General Hospital Songshan Branch, National Defense Medical Center, Taipei 114, Taiwan; letien54@yahoo.com.tw

**Keywords:** human conjunctival fibroblast, AR12286, TGF-β, α-SMA, SMAD, glaucoma filtration surgery

## Abstract

Scar formation can cause the failure of glaucoma filtration surgery. We investigated the effect of AR12286, a selective Rho-associated kinase inhibitor, on myofibroblast transdifferentiation and intraocular pressure assessment in rabbit glaucoma filtration surgery models. Cell migration and collagen contraction were used to demonstrate the functionality of AR12286-modulated human conjunctival fibroblasts (HConFs). Polymerase chain reaction quantitative analysis was used to determine the effect of AR12286 on the production of collagen Type 1A1 and fibronectin 1. Cell migration and collagen contraction in HConFs were activated by TGF-β1. However, compared with the control group, rabbit models treated with AR12286 exhibited higher reduction in intraocular pressure after filtration surgery, and decreased collagen levels at the wound site in vivo. Therefore, increased α-SMA expression in HConFs induced by TGF-β1 could be inhibited by AR12286, and the production of Type 1A1 collagen and fibronectin 1 in TGF-β1-treated HConFs was inhibited by AR12286. Overall, the stimulation of HConFs by TGF-β1 was alleviated by AR12286, and this effect was mediated by the downregulation of TGF-β receptor-related SMAD signaling pathways. In vivo results indicated that AR12286 thus improves the outcome of filtration surgery as a result of its antifibrotic action in the bleb tissue because AR12286 inhibited the TGF-β receptor-related signaling pathway, suppressing several downstream reactions in myofibroblast transdifferentiation.

## 1. Introduction

Glaucoma, the second leading cause of blindness after cataracts, results from the imbalanced production and elimination of aqueous humor, and increases intraocular pressure (IOP). High IOP is not the only factor causing glaucoma, but lowering IOP remains a principal clinical treatment [1]. If the recommended treatments do not achieve adequate IOP reduction with acceptable adverse effects, laser or incisional surgeries are indicated [2].

Trabeculectomy has been the standard surgical treatment for glaucoma since 1960, and it is the most common type of filtration surgery to reduce IOP in glaucoma [3,4]. The success of trabeculectomy principally depends on the filtering bleb during the postoperative wound-healing process. Excessive proliferation of fibroblasts or fibrosis in the subconjunctiva during the healing period causes tissue to scar, and leads subsequently to bleb failure [5,6].

Mitomycin C (MMC) and 5-fluorouracil (5-FU) have been used to counteract the proliferative activity of cells to prevent fibrosis after trabeculectomy [7,8,9,10]. MMC improves the success rate; however, bleb failure still occurs in certain cases because of unexpected fibrosis [11,12]. Furthermore, MMC has nonspecific cytotoxic effects that are associated with severe complications, such as blebitis, keratitis, bleb leakage, chronic hypotony, and endophthalmitis, caused by the untargeted destructive cellular toxicity of MMC. A study reported that 5-FU treatment complications include corneal erosion, bleb leakage, ruptures, cystic blebs, and hypotony [13]. Therefore, identifying safer and more broadly targeted antifibrotic agents that can be used postoperatively in addition to MMC and 5-FU is crucial to ensuring retention of bleb function.

Several chemical Rho kinase (ROCK) inhibitors were identified. Among them, fasudil has been approved for the treatment of vasospasm following subarachnoid hemorrhage. Another inhibitor of ROCK I, referred to as Y-27632, was reported to exhibit the ability to significantly reduce IOP in animals, alter cell shape, and alter the actin cytoskeleton of cultured human trabecular meshwork (TM) cells [14,15].

ROCK inhibitors were demonstrated to reduce IOP in rabbit and monkey models by increasing aqueous humor drainage through the primary outflow pathway in the eye, namely, the TM. Furthermore, ROCK inhibitors were demonstrated to increase ocular blood flow and enhance retinal ganglion cell survival after ischemic injury. The two mechanisms of ROCK inhibitors appear to reduce glaucoma [16]. Therefore, ROCK inhibitors have become a prominent novel approach to glaucoma treatment. TGF-β plays a crucial role in tissue fibrosis, including bleb fibrosis. ROCK inhibitors reduced TGF-β-induced epithelial–mesenchymal transition (EMT) in lens epithelial cells in vitro and in vivo, indicating that the Rho signaling pathway is involved in TGF-β-related functions [17].

Several studies reported that ROCK inhibitors reduce fibrotic formations in different tissue types, such as pulmonary [18], liver [19], and myocardial [20] tissue. Therefore, the antifibrotic effect of ROCK inhibitors may be useful in improving glaucoma filtering surgery by decreasing bleb fibrosis. In 2007, first-generation ROCK inhibitor Y-27632 was reported to have profound effects on human Tenon fibroblasts, and was effective in preventing fibroproliferation and scar formation in rabbit glaucoma surgery models [21].

The effects of decreasing IOP and antifibrotic activity, as induced by ROCK inhibitors, may have double the benefits in controlling IOP after glaucoma filtration surgery.

Recent research has led to the development of a series of novel ROCK inhibitors with enhanced potency within the low nanomolar range and improved kinase selectivity. AR12286 was demonstrated to possess single-digit nanomolar inhibitory activity against ROCK in enzymatic inhibition assays. Mechanism-of-action studies in monkeys demonstrated that AR12286 lowers IOP by increasing aqueous humor outflow through the TM [22]. In humans, AR12286 was effective in lowering IOP, and was determined to be safe for eyedrop use [23,24].

In the present study, we used AR12286 to evaluate the antifibrotic effect on cell migration inhibition, cell contractility, and myofibroblast transdifferentiation. Glaucoma filtration surgery was also investigated in rabbit models.

## 2. Materials and Methods

### 2.1. Rabbit Trabeculectomy Models 

Ocular trabeculectomies were performed in New Zealand white rabbits weighing 2–3 kg. The surgical operation was performed only on the right eye, and all surgeries and examinations were performed under general anesthesia. The rabbits were anesthetized with an intramuscular injection of 50 mg/kg ketamine and 10 mg/kg xylazine. A topical ophthalmic anesthetic using 0.5% proparacaine eye drops (Alcaine; Alcon, Puurs, Belgium) was also administered. After an eyelid speculum was used to retract the eyelids, a standard-sized 5-mm fornix-based conjunctival flap was created in the superior lateral quadrant of the eye at the limbus. Blunt dissection was applied to undermine the conjunctiva and Tenon’s capsule. A full-thickness scleral tunnel was then created using a 23-gauge needle that was inserted in the anterior chamber (AC) without touching the iridal blood vessels. Viscoelastic was injected through the needle to maintain the AC, and the needle was withdrawn. After entering the AC, the cannula was fixed on the sclera with a nonabsorbable 10-0 nylon suture material. The conjunctiva and Tenon’s capsule were closed in a single layer, using a nonabsorbable 10-0 nylon suture material attached to a BV needle in a continuous locking pattern. The formation of a filtering bleb was observed immediately after surgery. Topical AR12286 (presented from Aerie Pharmaceuticals, Inc., Bridgewater, NJ) at a dose of 0.5% and phosphate-buffered saline (PBS) were administered twice a day to the experiment and control groups, respectively, for seven postoperative days. The rabbit eyes were examined using routine clinical procedures, including IOP measurement. IOP of the rabbits was monitored every afternoon using an applanation tonometer (TonoVet; Icare Finland, Espoo, Finland). IOP was recorded before surgery, as the baseline, and on designated days after surgery. The average of three measurements was used as the IOP reading record. All experiment procedures were approved by the Animal Care and Use Committee of the National Defense Medical Center, and were performed according to the Association for Research in Vision and Ophthalmology Statement for the Use of Animals in Ophthalmic and Vision Research.

### 2.2. Histological Examination

Episcleral tissue at the site of the trabeculectomies was trimmed and grounded for histological analysis eight days after surgery. The eyeballs were enucleated, fixed in 10% neutral buffered formalin solution for 24 h, dissected at the equator, and embedded in paraffin. Anterior segments of the eyes containing the cornea, sclera, and conjunctiva were first cut at a sagittal orientation at 5 μm thickness, mounted on subbed slides, and dried. The sections were dewaxed in xylene, rehydrated in alcohol, and stained with hematoxylin, eosin, and Masson’s trichrome stain.

### 2.3. Cell Culture

Primary human conjunctival fibroblasts (HConFs) were obtained from ScienCell Research Laboratories (Carlsbad, CA, USA). Following the manufacturer’s instructions, HConFs were isolated from the conjunctiva of human eyes, dissected, and digested by enzymes. HConFs cultures were grown in fibroblast medium (ScienCell) supplemented with 5% fetal bovine serum (ScienCell), fibroblast growth supplement (ScienCell), and penicillin–streptomycin (100 U/mL and 100 mg/mL) solution (ScienCell). Primary HConFs were generated using an expansion culture of human Tenon’s explants propagated in Dulbecco’s modified Eagle’s medium (DMEM–F12; Gibco, Carlsbad, CA, USA) supplemented with 10% FBS (Gibco), 100 U/mL penicillin, and 100 μg/mL streptomycin (Sigma-Aldrich, St. Louis, MO, USA). Cells were maintained in the logarithmic growth phase. HConFs at passages 3 to 6 were used for all experiments. Experiments were replicated six times.

### 2.4. Cell Viability Assay

Cell viability assays were performed using the ready-to-use cell viability reagent 4-[3-(4iodophenyl)-2-(4-nitrophenyl)-2H-5-tetrazolio]-1,3-benzene disulfonate (WST-1; Roche Diagnostics, Indianapolis, IN, USA). After treatment for 24 h with various levels of AR12286 (Aerie Pharmaceuticals, Durham, NC 27703, USA) in serum-free medium, 10 μL of the WST-1 reagent was added to the medium in each well. Cells were incubated in a humidified atmosphere at 37 °C in 5% CO_2_/95% air for 1 h. The multitier plate was then shaken thoroughly for 1 min, and absorbance values were read at 450 nm. Background absorbance was measured from wells containing only the dye solution and culture medium. Cell viability data were collected from at least three experiments with at least six wells at each concentration in separate 96-well plates. The mean optical-density values of the untreated controls were assigned values of 100%. Results are expressed as the percentage of optical density of the treated cells relative to the optical density of the untreated controls.

### 2.5. Western Blot Analysis

For Western blot analysis, cells were treated with 10 ng/mL TGF-β1 (Pepro Tech, New York, NY, USA) for 24 h in the absence and presence of AR12286 at 250 or 500 nM. The treated and untreated cells were washed with PBS, harvested by scraping, and centrifuged at 1000× *g*. Cell pellets were resuspended and sonicated in a cold lysis buffer (EDTA-free PROPREPTM Protein Extraction Solution; iNtRON Biotechnology, Inc., Seoul, Korea). Lysates were then centrifuged for 10 min at 12,000× *g*, and protein concentration in the clear supernatant was determined using the BCA protein assay kit (Pierce, Rockford, IL, USA). Lysates (20 μg) were resolved by 10% SDS-PAGE and transferred to PVDF membranes. Membranes were blocked with 5% (*w/v*) milk for 1 h at room temperature and incubated for 1 h at room temperature with 1:1000 dilutions of antibodies against GAPDH (Millipore, Rockland, NY, USA), α-SMA (Sigma-Aldrich), SMAD2, and phosphorylated SMAD2 antibodies (Cell Signaling Technology, Danvers, MA, USA). Membranes were washed and incubated with horseradish peroxidase-conjugated secondary antibodies (1:1000; Jackson ImmunoResearch Laboratories, West Grove, PA, USA) for 1 h at room temperature, and proteins were visualized using an enhanced chemiluminescence procedure (enhanced chemiluminescence reagent; Millipore, Billerica, MA, USA).

### 2.6. Migration Assays

Cell migration was measured using a modified Boyden chamber assay as previously described [25]. Briefly, HConFs cells were seeded at a density of 5 × 10^4^ cells/well in the upper chamber with an 8 μm transwell pore (Millipore). The lower chamber was filled with 0.1% FBS–DMEM–F12 medium containing 10 ng/mL TGF-β1. After incubation for 5 h, the inserts were washed with PBS, fixed with cold methanol (4 °C) for 10 min, and counterstained with hematoxylin for 20 min. Migration cell numbers were counted using phase contrast microscopy. Four randomly selected fields were calculated for each insert.

### 2.7. RNA Isolation and Quantitative Real-Time Polymerase Chain Reaction

Expression of Type I collagen and fibronectin genes in HConFs were investigated at the mRNA level using real-time polymerase chain reaction (RT-PCR). Total RNA from HConFs was isolated (TRIzol Reagent; Invitrogen-Gibco, Grand Island, NY, USA) according to the manufacturer’s instructions. RT-PCR of *COL1A1* and *FN 1* was performed using the TaqMan method and primers for collagen Type I alpha 1 (COL1A1, Hs00164004_m1, Applied Biosystems, CA, USA) and fibronectin 1 (FN 1, Hs00365052_m1, Applied Biosystems, CA, USA). The amplification reactions were performed in duplicate using 96-well plates with 0.5 μL of primers and probe, 5 μL of master mix, 2.5 μL of cDNA, and 2.0 μL of diethylpyrocarbonate-treated water. The thermal cycling profile consisted of an initial temperature of 50 °C for 2 min, denaturation at 95 °C for 10 min, and then 40 successive cycles at 95 °C for 15 s and 60 °C for 1 min. The quantities of the target mRNA were calculated relative to the endogenous control expression and values from the control group by using the 2−ΔΔCt method. The mRNA expression levels were normalized using GAPDH expression as an endogenous housekeeping gene.

### 2.8. Collagen Matrix Contraction Assay

Cell-populated three-dimensional collagen matrix contraction was analyzed using a method described by Mazure and Grierson [26] with minor modifications. Type I collagen from rat tails (Sigma-Aldrich) was dissolved in 0.1% acetic acid and stored at 4 °C overnight. The wells of a 24-well plate were preincubated overnight with 2% FBS to block nonspecific binding. HConF cells were resuspended at a density of 1 × 10^6^ cells/mL in DMEM–F12. The cell suspension was mixed with 5.0 mL of 3 mg/mL collagen (Sigma-Aldrich) and 3.0 mL of concentrated serum-free MEM containing glutamine, antibiotics (100 U/mL penicillin and 100 μg/mL streptomycin), and 391 μL of 1 M NaOH. The cell–collagen mixture was seeded in a 24-well plate (350 μL/well) and incubated at 37 °C for 1 h to allow for polymerization. Collagen gels were detached from the bottoms of the wells after 1.5 h, and the matrices were floated in 1 mL DMEM–F12 containing 10% FBS. After 24 h, the medium was aspirated, and the gels were washed with serum-free DMEM–F12 and incubated at 37 °C for 3 days in serum-free DMEM–F12 containing 10 ng/mL TGF-β1 with or without AR12286. The medium was replaced every other day. The baseline contractions were calculated in collagen gels without HCF cells. The surface area of each matrix was observed, recorded, and digitally measured using an LAS-3000 CCD camera (Fujifilm, Düsseldorf, Germany) from Days 1 to 3. The percentage of gel contraction was calculated as (gel size at Days 1–3/gel size at Day 0) × 100.

### 2.9. Statistical Analysis

Data are presented as means ± standard errors of the mean. Each result is representative of at least six independent experiments. Normally distributed continuous variables were compared using one-way analysis of variance. When significant difference between groups was demonstrated, multiple comparisons of means were performed using the Student–Newman–Keuls procedure. All statistical assessments were two-sided and evaluated at the 0.05 level of statistical significance.

## 3. Results

### 3.1. Evaluation of AR12286 in Rabbit Trabeculectomy Models

**Figure 1:**
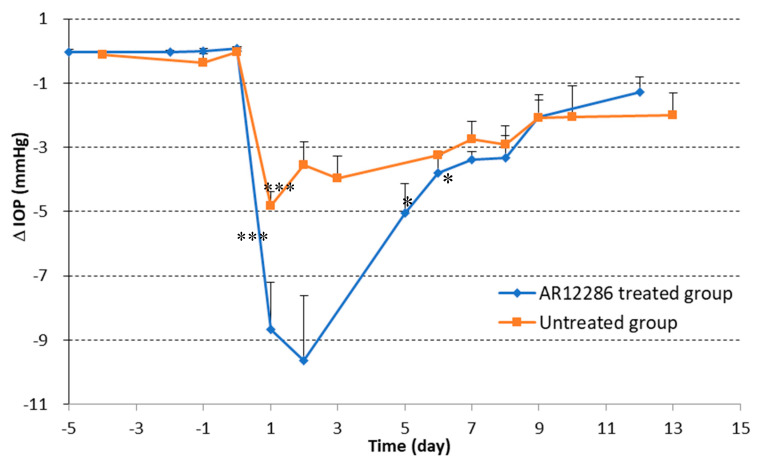
Effect of AR12286 on intraocular pressure (IOP) maintenance in rabbit models of trabeculectomy. IOP changes over entire experimental period are illustrated. Compared with IOP from the same rabbits prior to surgery (baseline), IOP levels in both groups were at lower levels throughout the entire experiment period. ∆IOP is the difference between IOP of surgical and nonsurgical eyes. ∆IOP in AR12286 group rabbits (trabeculectomy with AR12286 treatment) exhibited significantly lower level at Days 1 (*p* < 0.001), 2 (*p* < 0.001), 6 (*p* < 0.05), and 7 to 8 (*p* < 0.05) than IOP from control group (trabeculectomy only). Asterisks indicate significantly different responses (*, *p* < 0.05; ***, *p* < 0.001).

This study was designed to evaluate the antifibrotic effect of AR12286 in an animal model and the lowering of IOP. We applied topical agents (with or without AR12286) twice daily on the surgical wound site in rabbit models of trabeculectomy. IOP is a critical parameter for the evaluation of the outcome of glaucoma surgery. IOP measurements were performed on the designated days to evaluate the effect of AR12286 on IOP after glaucoma surgery. The results for the value of ∆IOP, which reflects IOP differences between the surgical and nonsurgical eye, are presented in Figure 1. Before surgery, no significant difference in IOP was observed between the two groups. In this experimental design, the control group was assessed after 13 days, whereas the treated group was assessed after 12 days. In both groups, IOP reduction caused by the surgery was maintained at a lower level than the IOP from the same animals before surgery for the entire experiment period after surgery. Furthermore, the IOP of rabbits in the AR12286 group (trabeculectomy with AR12286 treatment) was maintained at a significantly lower level at Days 1 (*p* < 0.001), 2 (*p* < 0.001), 6 (*p* < 0.05), and 7 to 8 (*p* < 0.05) than the IOP of the control group (trabeculectomy only; Figure 1). 

The control group exhibited IOP reduction of 3 to 5 mmHg prior to Day 8; however, the group treated with AR12286 exhibited reduction in IOP of 5 to 10 mmHg prior to Day 5, and a similar reduction relative to the control after Day 5.

**Figure 2:**
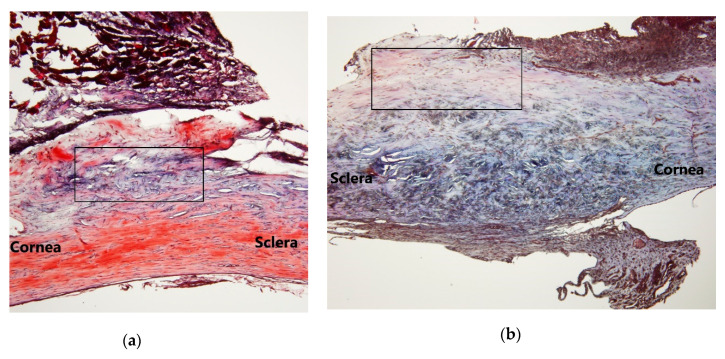

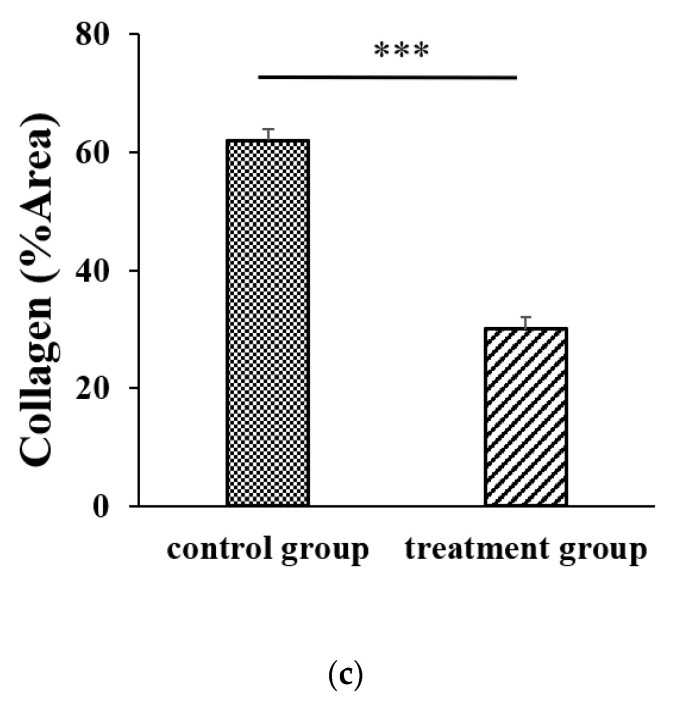
Fibrotic effects of AR12286 on post-trabeculectomy bleb tissue. Histological examination of bleb tissue from (**a**) control group (without AR12286) and (**b**) treatment group (injected with AR12286) of experimental rabbit trabeculectomy models. Collagen deposition (blue) examined using Masson’s trichrome stain (**a**). On Day 8 post-surgery, less intense blue stain was evident in the bleb tissue compared with that in the control group, indicating that AR12286 reduced collagen deposition in bleb tissue. (**c**) Ratios of collagen-fiber areas to conjunctival and scleral lesions in control and treated eyes. Values represented as means ± SEM (*n* = 6). *** indicates *p* < 0.001 compared with controlled eyes.

Masson’s trichrome staining was used to identify differences in collagen production in subconjunctival tissue. With respect to collagen expression in the site on Day 8 (Figure 2), shortened focal perpendicular sequences of collagen fibers were observed in the control group (Figure 2a), and much looser and multidirectionally oriented hypocellular connective tissue and rare collagen deposition were observed in the group treated with AR12286. Fibrosis inhibition was demonstrated by reduced collagen deposition in the subconjunctival space. Along with inducing further IOP reduction, these results demonstrated that AR12286 could extend the IOP reduction effect in the examined trabeculectomy model by alleviating fibrosis.

### 3.2. Viability of Using AR12286 against Human Conjunctival Fibroblasts

**Figure 3:**
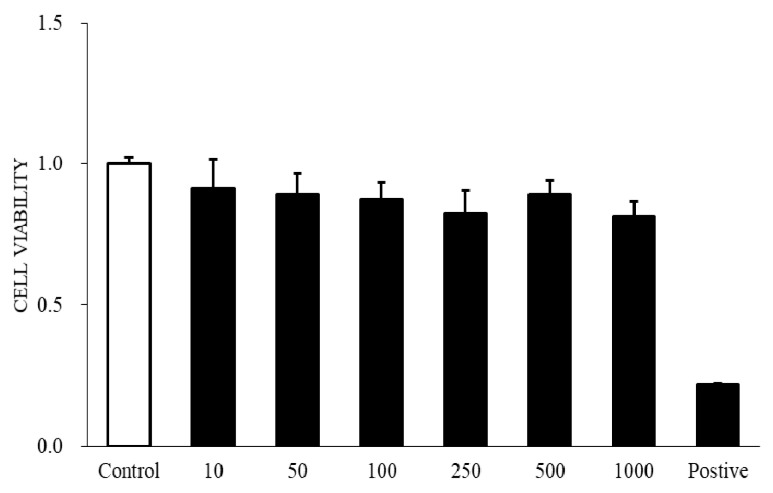
Viability of AR12286 used against human conjunctival fibroblasts (HConFs). WST-1 assay applied to assess cell viability. Cells incubated with AR12286 for 24 h in concentrations from 10 to 1000 nM. AR12286 levels lower than 1000 nM displayed no obvious harmful effects on HConFs. All data expressed as means ± SEM (*n* = 6).

Cell viability was determined using the WST-1 assay treated with AR12286 in HConFs 24 h later. Results are illustrated in Figure 3. Lower cytotoxicity to HConFs was observed for AR12286 concentrations of 10, 50, 100, 250, 500, and 1000 nM. AR12286 concentrations of 250 and 500 nM were selected for further study.

### 3.3. Cell Migration Effect Inhibited by AR12286

**Figure 4:**
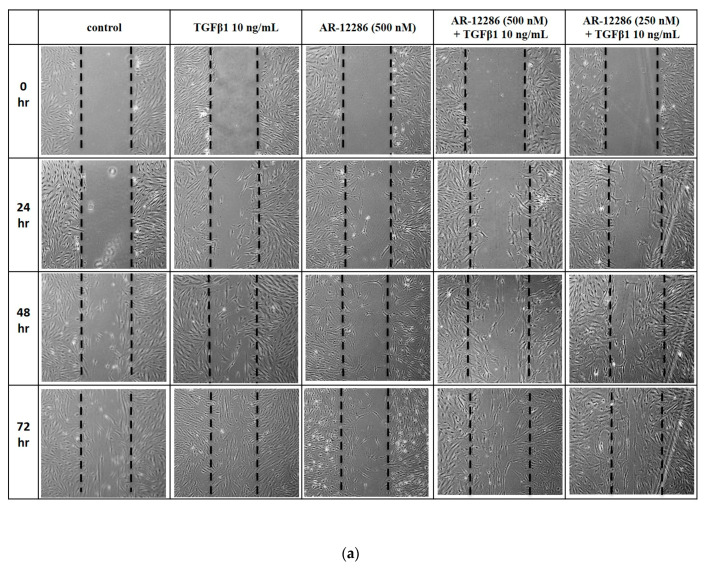

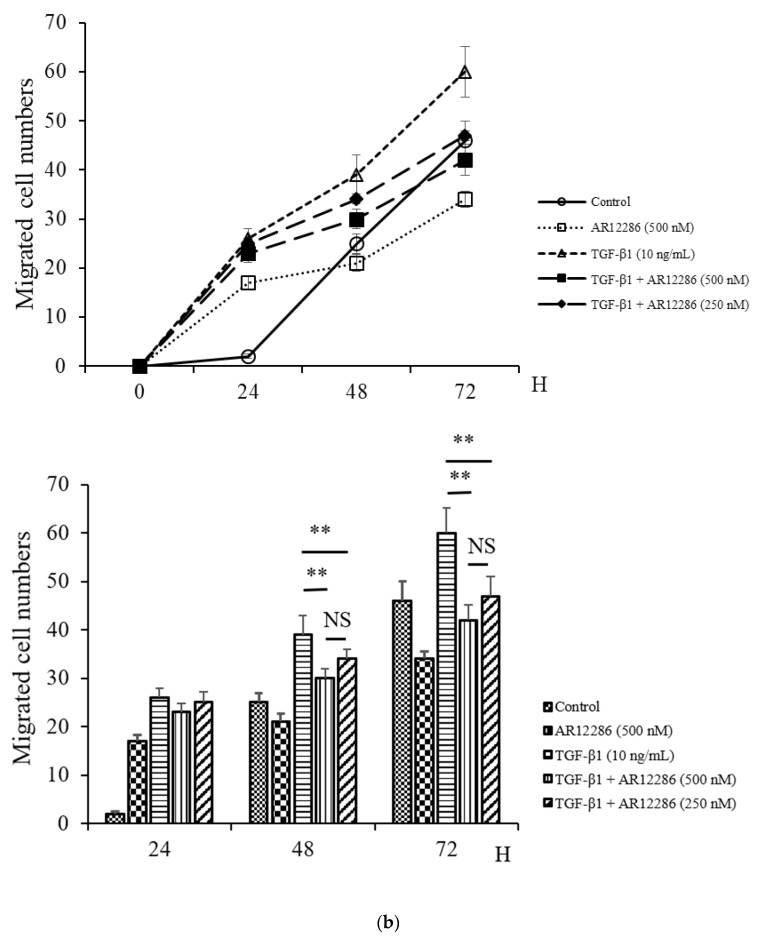
Cell migration inhibited by AR12286. Cell-free linear wound presented when confluent cultures were scraped with a pipette tip. Medium was individually replaced with fresh cultured medium in the following cultures: control, TGF-β1-induced, AR12286-treated, TGF-β1-induced with 250 nM treatment of AR12286, and TGF-β1-induced with 500 nM treatment of AR12286. (**a**) Cell migration into the wound area photographed after incubation for 24, 48, and 72 h; (**b**) number of migrated cells was determined. Dotted lines, edges of migrated cells. All data expressed as means ± SEM (*n* = 6). Asterisks indicate significantly different responses (**, *p* < 0.01), and NS signifies no significant difference.

Fibroblast migration to the wound site plays a pivotal role in the wound-healing process, which is stimulated by TGF-β1. To assess the effect of AR12286 on TGF-β1-induced cell migration of HConFs, a modified Boyden chamber assay was used. After 24, 48, and 72 h, cell migration results were measured. As illustrated in Figure 4, HConFs treated with TGF-β1 displayed significantly higher levels of cell migration after 24 (*p* < 0.001), 48 (*p* < 0.01), and 72 (*p* < 0.05) h. 

After 24 h, HConF migration induced by TGF-β1 was 10-fold higher than that of the control. This migration was not inhibited by 250 and 500 nM of AR12286. However, at 48 and 72 h, cell migration of HConFs induced by TGF-β1 was inhibited by both 250 (*p* < 0.01) and 500 nM (*p* < 0.01) concentrations of AR12286.

### 3.4. Effect of AR12286 on α-SMA Expression

**Figure 5:**
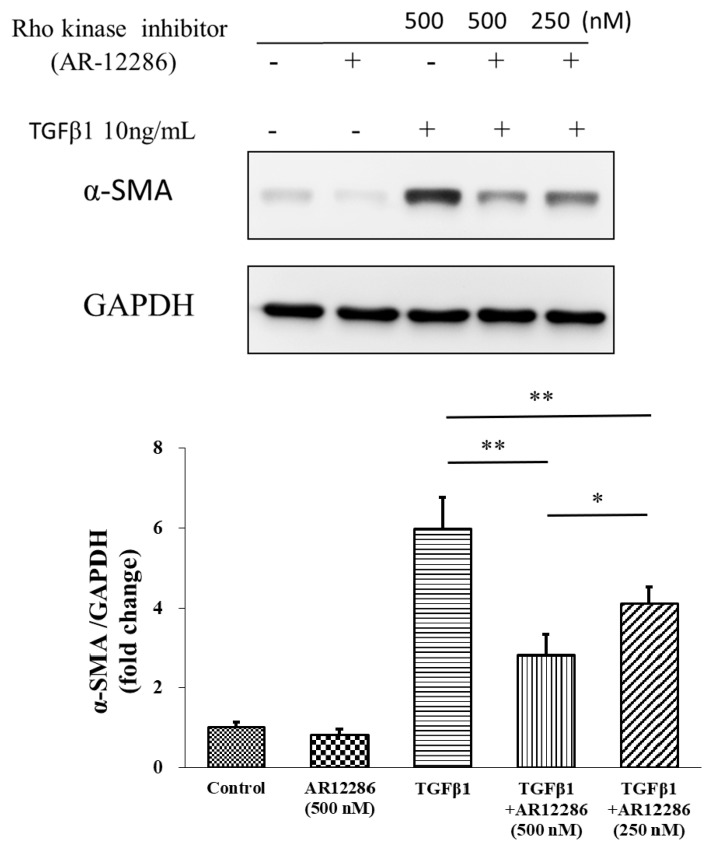
Effect of AR12286 on α-SMA expression. Five groups compared to evaluate α-SMA expression; 250 and 500 nM AR12286 applied to affect α-SMA expression in HConFs. HConF cells co-cultured with TGF-β1 had significantly higher α-SMA expression than the control or 500 nM AR12286 cells did. Other results demonstrated that α-SMA expression could be attenuated by AR12286 in a dose-dependent manner in 250 and 500 nM (*p* < 0.05). Data presented as means ± SEM (*n* = 6). Asterisks indicate significantly different responses (*, *p* < 0.05; **, *p* < 0.01).

In this experiment, five groups were compared to assess α-SMA expression. The effects of 250 and 500 nM AR12286 on α-SMA expression changes in HConFs were investigated. As illustrated in Figure 5, HConFs co-cultured with TGF-β1 exhibited significantly higher α-SMA expression than the controls or the HConFs co-cultured with an AR12286 concentration of 500 nM did. Our results further demonstrated that α-SMA expression could be attenuated by AR12286 in a dose-related manner at 250 and 500 nM (*p* < 0.05).

### 3.5. Effect of AR12286 on Gel Contraction

**Figure 6:**
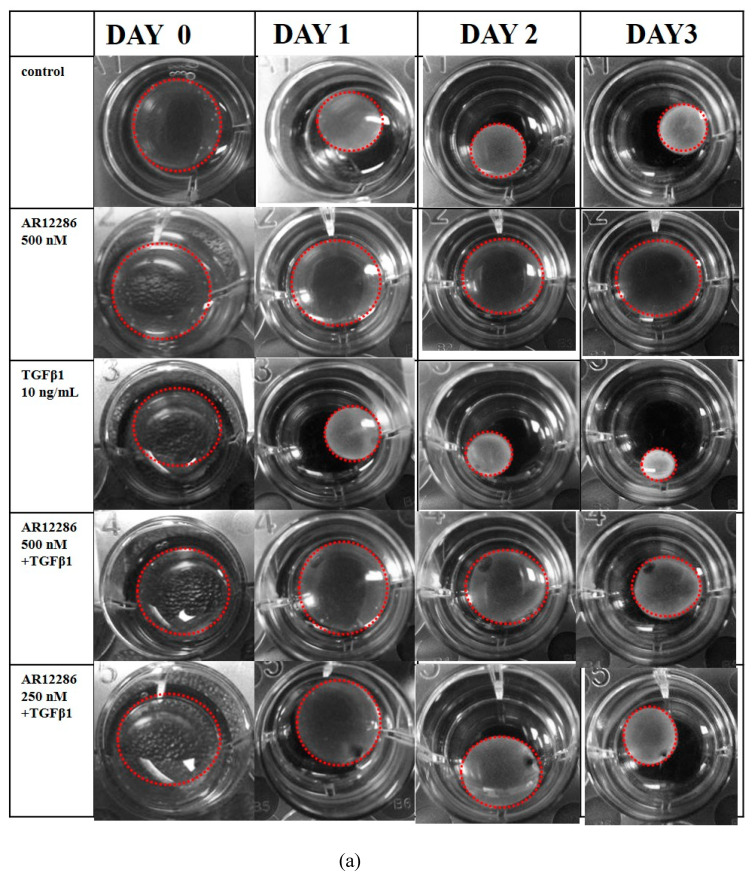

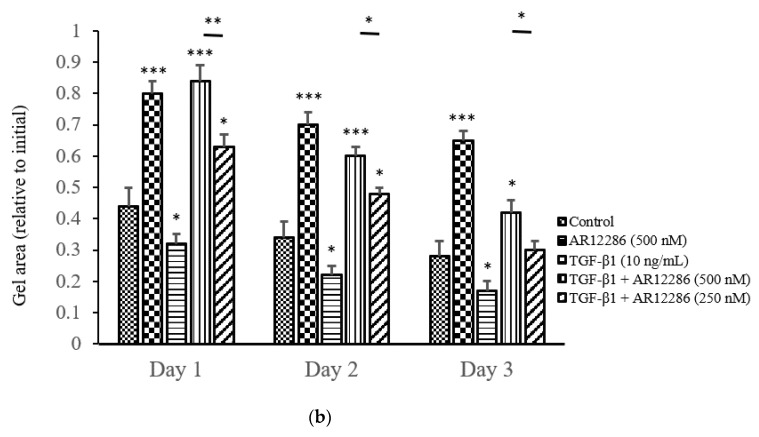
Effect of AR12286 on gel contraction. (**a**) Control, AR12286-treated, TGF-β1-induced and 250 nM AR12286-treated, and TGF-β1-induced and 500 nM AR12286-treated collagen gels incubated for up to 3 days. (**b**) Contracted extent of collagen gels mediated by HConFs expressed as gel size based on initial area. Gel size (relative to initial) measured in absence (control) or presence of AR12286 at 250 and 500 nM. Control group exhibited gradual decrease in gel-size ratio: 0.43 (Day 1), 0.35 (Day 2), and 0.25 (Day 3). TGF-β1-treated group exhibited significantly more intense contraction than the control group did from Days 1 to 3 (*p* < 0.05). Data presented as means ± SEM (*n* = 6). Single asterisk indicates statistical difference compared with control group. Asterisks indicate the following significantly different responses: *, *p* < 0.05; **, *p* < 0.01; and ***, *p* < 0.001.

The contraction of floating collagen gels was considered to mimic the reorganization of the collagenous matrix during tissue development and healing. To evaluate the effect of AR12286 on TGF-β1-induced collagen gel contraction, we measured the area of the gels using freshly polymerized collagen matrices containing HConFs incubated with TGF-β1 in the absence or presence of AR12286 (Figure 6a). Gel-area ratio (relative to initial) was used to evaluate the contraction results. The control group exhibited a gradual reduction in gel-size ratio of 0.43 (Day 1), 0.35 (Day 2), and 0.25 (Day 3). The TGF-β1 treatment group displayed significantly more intense contractions than the control group did from Days 1 to 3 (*p* < 0.05). Results further demonstrated that the contraction induced by TGF-β1 could be inhibited by AR12286. The contraction in response to TGF-β1 was significantly counteracted by the presence of AR12286 in a dose-related manner (Figure 6b).

### 3.6. Effect of AR12286 on COL1A1 and FN 1 Gene Expression

**Figure 7:**
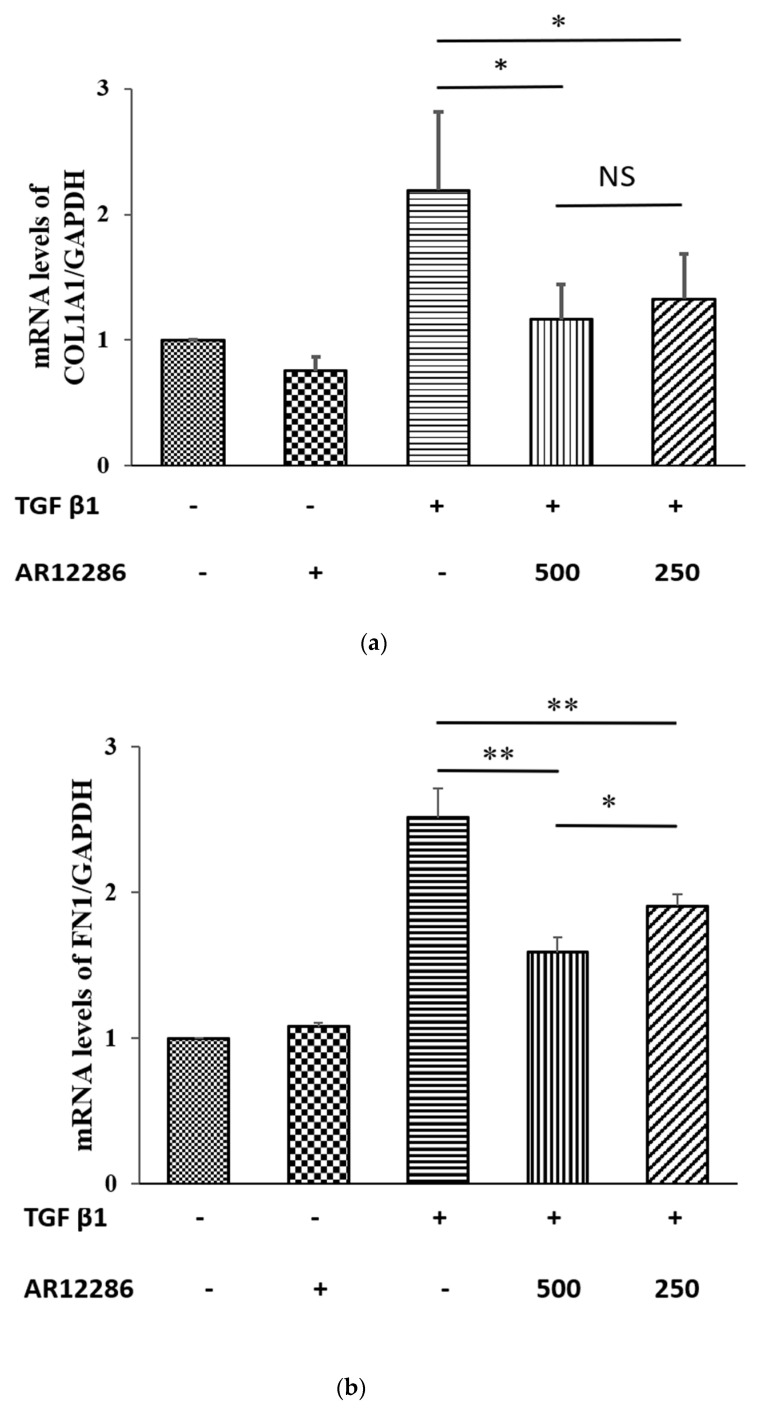
Effect of AR12286 on (**a**) COL1A1 and FN 1 (**b**) gene expression. AR12286 did not affect COL1A1 and FN 1 expression. However, TGF-β1 significantly increased COL1A1 and FN 1 production. TGF-β1-induced production of COL1A1 and FN 1 inhibited by AR12286. Data presented as means ± SEM (*n* = 6). Asterisks indicate significantly different responses: **, *p* < 0.01; *, *p* < 0.05. NS signifies no significant difference.

COL1A1 encodes the major component of Type I collagen, which is the fibrillar collagen found in most connective tissue, including bleb tissue. FN 1 encodes fibronectin, which is also present in fibrotic matrices, is required for collagen matrix assembly. mRNA levels of COL1A1 and FN 1 were measured in AR12286-treated TGF-β1-induced HConFs. To assess the effect of AR12286 on TGF-β1-induced COL1A1 and FN 1 production in HConFs, mRNA levels in the presence and absence of AR12286 were compared. Results revealed that the expression of COL1A1 and FN 1 genes was significantly increased in HConFs treated with TGF-β1 (Figure 7) compared with that in the control group. However, higher expression of COL1A1 and FN 1 in these cells was significantly inhibited after the addition of either 250 or 500 nM AR12286 (*p* < 0.01). These two doses of AR12286 had similar downregulating expressions.

### 3.7. Effect of AR12286 on SMAD Deactivation

**Figure 8:**
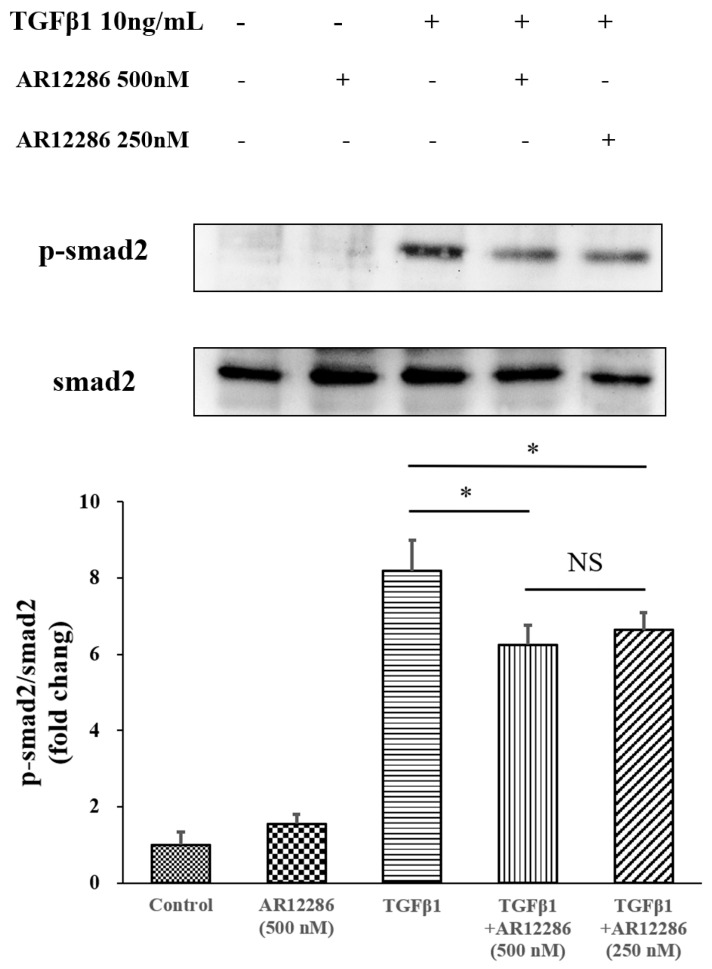
Effect of AR12286 on SMAD deactivation. SMAD2 expression and phosphorylation are illustrated. Serum-starved HConFs pretreated with either 250 or 500 nM AR12286 for 24 h. Cells were treated with TGF-β1 for 60 min, and cell lysates were immunoblotted with antiphosphorylated and antitotal antibodies. Protein loading normalized with GAPDH antibodies. Compared with ratios of the control group, those of *p*-SMAD2 to total SMAD2 increased after TGF-β1 stimulation, and decreased after treatment with 250 or 500 nM AR12286. Ratio presented as means ± SEM (*n* = 6). Asterisks indicate significantly different responses: *, *p* < 0.05. NS signifies no significant difference.

SMADs act as downstream transcription factors that propagate TGF-β receptor signals. We evaluated the effect of AR12286 on SMAD phosphorylation. HConFs were treated with TGF-β1 in the presence or absence of AR12286, and Western blot analysis with antiphosphospecific and anti-SMAD2 antibodies was performed. TGF-β1 treatment caused an elevation in the levels of the phosphorylated forms of SMAD2 in HConFs. This phosphorylation was reduced by cell pretreatment with either 250 or 500 nM concentrations of AR12286 (*p* < 0.01; Figure 8).

## 4. Discussion

Adequately regulating the healing process at the surgical site of glaucoma filtration surgery (GFS) remains a challenge. Conjunctival wound healing is required to prevent bleb leakage, and fibrosis reduction in both the subconjunctival space and drainage pathway is also required to increase the success rate of GFS. In the present study, we demonstrated that the myofibroblast transdifferentiation of HConFs induced by TGF-β1 was significantly inhibited by AR12286, and the outcome of IOP reduction was enhanced by AR12286 in the animal model.

AR12286, which is a highly selective ROCK inhibitor, was developed by screening a collection of water-soluble amino-isoquinoline amides that are both stable and active in altering the shape of TM cells [23]. Clinical trial Phase 1 and 2 studies of AR12286 were conducted to explore its potential ability to lower IOP [24,27]. Certain drugs with different mechanisms, such as α-lipoic acid (antioxidant), trichostatin A (histone deacetylase inhibitor), and rosiglitazone (peroxisome proliferator-activated receptor γ (PPARγ)-selective agonist), were investigated to improve trabeculectomy surgery [28,29,30]. In the present study, the antifibrotic ability of AR12286 was evaluated in HConFs in vitro and in vivo in rabbits.

First, we applied topical AR12286 in an animal study to evaluate whether AR12286 enhanced the efficiency of IOP reduction. Our findings indicated that AR12286 administration significantly lowered IOP during eye filtration surgery compared with treatment without AR12286 (Figure 1). This IOP reduction phenomenon was consistent with previous findings on Y-27632 [21]. However, AR12286 reportedly lowered IOP by altering the TM structure. Collagen expression could indicate fibrosis formation and cause bleb failure. Our study results (Figure 2) demonstrated that collagen deposits in the bleb fibrosis site were significantly lower at postoperative Day 8 with topical administration of AR12286 in rabbits. This result suggested that AR12286 treatment lowers fibrotic reactions in the subconjunctival site, and reduced scar formation may enhance the success rate of filtering surgeries. Increased efficiency in reducing IOP post-surgery resulted from improving TM resistance with AR12286 and from the antifibrotic effect of AR12286, which reduced collagen deposition at the bleb site.

Fibroblast migration to the wound site is a prerequisite for wound healing. Fibroblasts are involved in the construction and remodeling of scar tissue [31]. We evaluated the effect of AR12286 on TGF-β1 in fibroblast migration. AR12286 was demonstrated to have no toxic effect on cell viability at concentrations of 10 to 1000 nM. Fibroblast cell motility tests revealed that AR12286 treatment failed to induce cell migration compared with that in the control group. After 48 and 72 h, AR12286 suppressed HConFs TGF-β1-induced migration to the wound site (Figure 4). The effect of cell migration suppression was dose-related. AR12286 inhibited conjunctival fibroblast migration; this is consistent with previous findings on H-1152P [32]. In a study by Tura et al., H-1152P at 1 and 10 μM reduced cell migration after 48 h (20%–35%) in a dose-dependent manner.

TGF-β was induced to promote wound healing, recruit fibroblasts, and increase fibroblast proliferation [33]. Fibroblasts subsequently differentiate into the contractile phenotype, termed myofibroblasts, during TGF-β-induced scar formation [34]. To mimic TGF-β secretion after glaucoma surgery and assess the ability of AR12286 to block the effect of TGF-β, HConFs were stimulated before AR12286 treatment, and collagen contractility was evaluated to assess the effect of AR12286 on myofibroblast transdifferentiation. Collagen contraction was not affected by AR12286 treatment, but the extent of collagen induction by TGF-β1 was reduced by AR12286. Gel contraction alleviated by AR12286 was dose-related. These results are similar to findings from a study by Pitha et al. [35]. Three types of ROCK inhibitors, namely, H1152, Y-27632, and fasudil, were applied in scleral fibroblasts. H1152 and Y-27632 concentrations of 0.4 to 10 μM could reduce gel contraction induced by TGF-β1 in a dose-dependent manner; fasudil achieved a similar effect with concentrations of 1 to 25 μM.

Myofibroblasts are sometimes referred to as “activated” fibroblastic cells. The prominent microfilament bundles of myofibroblasts form stress fibers that enable contraction of the cell, thus remodeling the adjacent extracellular matrix (ECM) [36]. Incorporating α-SMA, which is a characteristic of the myofibroblast, into cellular stress fibers significantly increases the contractile activity of myofibroblasts, and is a key marker of the myofibroblastic phenotype [37]. AR12286 reduced TGF-β1-induced α-SMA expression. In our study, 250 and 500 nM concentrations of AR12286 reduced α-SMA expression in a dose-dependent manner. Ripasudil (a ROCK inhibitor) similarly reduced α-SMA expression [38]. In a study by Futakuchi et al., HConFs were treated with 25 or 50 μM ripasudil prior to TGF-β2 stimulation, and α-SMA expression was significantly reduced in a dose-dependent manner.

The ECM components synthesized by cells alter as wound healing progresses. Collagen is a component of the ECM and a key protein in fibrosis. Type I collagen constitutes the main collagen of the scar, and COL1A1 gene encodes the major component of Type I collagen. In our study, TGF-β1-induced increase in the expression of COL1A1 was attenuated by pretreatment with AR12286. These data were consistent with findings indicating that Y-27632 inhibited TGF-β1-induced production of Type I collagen mRNA by Tenon fibroblasts [39]. Zhang et al. revealed that Y-27632 could reduce collagen mRNA levels when elevated by TGF-β. Collagen level after treatment with 750 μM of Y-27632 was five times lower than that of the control group. Moreover, Y-27632 concentrations of 6 to 30 μM exhibited dose-related reductions in collagen after treatment with TGF-β.

TGF-β signaling is essential for wound healing, which includes both nonspecific scar formation and tissue-specific regeneration. TGF-β isoforms include TGF-β1, TGF-β2, and TGF-β3, which are involved in canonical (SMAD) and noncanonical TGF-β signaling pathways. TGF-β1 induces the transition of fibroblasts into myofibroblasts. Myofibroblasts, as characterized by the expression of α-SMA, play a crucial role in wound healing, fibrosis formation, contraction, and connective tissue remodeling. In the canonical pathway, TGF-β1 binds to TGF-β receptor 2 (TGFR2), which then recruits and activates TGFR1. The active TGFR1 then phosphorylates SMAD2 and SMAD3, which form complexes with SMAD4 that translocate to the nucleus and induce transcription of profibrotic molecules, including α-SMA and collagen Type I proteins [40].

The TGF-β downstream signal pathway in SMAD pathways was also evaluated. TGF-β1 binds to TGFR2, which activates TGFR1. Phosphorylated SMAD2 and SMAD3 form complexes with SMAD4 and translocate into the nucleus for the next transcription. We further evaluated the SMAD2 pathway. AR12286 reduced the phosphorylated SMAD2 expression that is induced by TGF-β1. These findings differ from findings reported by Meyer-ter-Vehn et al. (2006) and Ibrahim et al. (2019) [3,41]. Meyer-ter-Vehn et al. explored the p38-dependence of TGF-β-induced myofibroblast transdifferentiation and provided a mechanism for the effect of H1152 on α-SMA expression. Ibrahim et al. demonstrated that Y-27632 inhibited MAPK activation (ERK, p38, JNK) in the early response of the MAPK-signaling pathway.

Studies demonstrated that ROCK inhibitors reduce fibrosis through non-SMAD pathways. However, our findings indicated the involvement of the SMAD pathway; this is consistent with our previous study, in which silibinin was applied. To further understand the mechanisms at play, an investigation is required into the role of the pathway in scar formation after glaucoma surgery and AR12286 modulation mechanisms.

ROCKs are associated with actin stress fibers and focal adhesions. Furthermore, ROCKs are considered to be crucial for cell migration, PTEN stimulation, cell-cycle control, membrane blebbing, and neurite retraction [42,43]. ROCKs have pleiotropic functions, including the regulation of cellular contraction, motility, morphology, polarity, cell division, and gene expression. Pharmacological effects of ROCKs demonstrate their involvement in various diseases, such as pulmonary hypertension, vasospasm, nerve injury, and glaucoma. Therefore, ROCKs are considered to be a potential therapeutic target [42]. Since the early 2000s, several inhibitor molecules have been developed with different relative potencies and biological functions [44].

Our in vitro and in vivo findings revealed that local adjunctive treatment with AR12286 in filtering surgeries may reduce scarring in blebs through the inhibitory effect of AR12286 on TGF-β1.

This study is limited in that it did not study other signaling pathways, various concentrations, or other agents for antifibrosis investigated in the in vitro study. Advanced analytical techniques, such as fluorescence immunocytochemistry, could be applied to evaluate the specific arrangement of fibroblast cytoskeleton and α-SMA fibers. More comprehensive panels of different genes implicated in the fibrotic process should be assessed in fibroblasts using different experiment conditions. Bleb-survival assessment should be evaluated in animal studies to demonstrate the increased success rate of trabeculectomy after treated agents. The study still requires other tissue analyses, such as measuring the differences of α-SMA-positive profibrotic myofibroblasts and the expression of TGF-β1 in vivo. In the SMAD pathway, activated TGF-β receptor 1 phosphorylates SMAD2 and SMAD3. SMAD4 is then recruited and the complex (SMAD2/3) is translocated to the nucleus. Thus, attenuated SMAD2 phosphorylation was considered in this study to implicate the induction of the signal inhibition of the SMAD pathway. In future studies, we will consider more different SMAD transduction signals to improve the SMAD pathway outcome.

AR12286 had been used to reduce IOP related to glaucoma by TM cell mechanisms, but this study was the first to investigate its antifibrotic effects after GFS. Further research is required to explore the involved mechanism and efficacy. Compared with treatment using other ROCK inhibitors, AR12286 treatment requires lower concentrations (250 and 500 nM). A study reported that 5 to 10 μM ROCK inhibitors were required for myofibroblast differentiation [35,41]. However, drug concentrations may prove to be a limitation of AR12286.

## 5. Conclusions

An antifibrotic effect of AR12286, whereby it reduced the production of collagen, was demonstrated in the rabbit model. AR12286 effectively inhibited TGF-β1-induced myofibroblast transdifferentiation, cell migration, and collagen contractility. Furthermore, SMAD2 phosphorylation was triggered by TGF-β1 and inhibited by A12286. TGF-β was the major factor in the failure of filtering blebs; thus, ROCK inhibitors such as AR12286 could be candidates that suppress TGF-β pathways and serve as an adjunctive agent to reduce the failure rate of glaucoma filtering surgeries.

Compared with other Rho kinase inhibitors, AR12286 is highly selective. Therefore, AR12286 may be a more attractive candidate for glaucoma filtration surgery due to its lower required dose and fewer side effects.
